# Taxifolin Targets PI3K and mTOR and Inhibits Glioblastoma Multiforme

**DOI:** 10.1155/2021/5560915

**Published:** 2021-08-20

**Authors:** Weiqi Yao, Hongyun Gong, Heng Mei, Lei Shi, Jinming Yu, Yu Hu

**Affiliations:** ^1^Department of Hematology, Union Hospital, Tong Ji Medical College, Hua Zhong University of Science and Technology, Wuhan, Hubei, China; ^2^Department of Radiation Oncology, Shandong Cancer Hospital Affiliated to Shandong University, Shandong Academy of Medical Science, Jinan, China; ^3^Cancer Center, Renmin Hospital of Wuhan University, Wuhan, Hubei, China; ^4^Fifth Medical Center of PLA General Hospital, National Clinical Research Center for Infectious Diseases, Beijing, China; ^5^Shandong Academy of Medical Science, Jinan, China

## Abstract

Glioblastoma multiforme (GBM), the most common malignant primary brain tumor, has a very poor prognosis. With increasing knowledge of tumor molecular biology, targeted therapies are becoming increasingly integral to comprehensive GBM treatment strategies. mTOR is a key downstream molecule of the PI3K/Akt signaling pathway, integrating input signals from growth factors, nutrients, and energy sources to regulate cell growth and cell proliferation through multiple cellular responses. mTOR/PI3K dual-targeted therapy has shown promise in managing various cancers. Here, we report that taxifolin, a flavanone commonly found in milk thistle, inhibited mTOR/PI3K, promoted autophagy, and suppressed lipid synthesis in GBM. *In silico* analysis showed that taxifolin can bind to the rapamycin binding site of mTOR and the catalytic site of PI3K (p110*α*). In *in vitro* experiments, taxifolin inhibited mTOR and PI3K activity in five different glioma cell lines. Lastly, we showed that taxifolin suppressed tumors in mice; stimulated expression of autophagy-related genes LC3B-II, Atg7, atg12, and Beclin-1; and inhibited expression of fatty acid synthesis-related genes C/EBP*α*, PPAR*γ*, FABP4, and FAS. Our observations suggest that taxifolin is potentially a valuable drug for treating GBM.

## 1. Introduction

Glioblastoma multiforme (GBM) is the most frequently occurring malignant tumor of the central nervous system (CNS) [1] and is notoriously treatment-resistant [2]. Among glial tumors, GBM is the most malignant and is characterized by poor prognosis and short median survival time for both pediatric and adult patients [3–5]. Standard therapies are inadequate and treatment side-effects can leave patients suffering severe morbidity [6]. The biology of GBM is complex and multiple signaling pathways have been implicated in GBM pathogenesis [7, 8]. Significant effort has thus been devoted to identifying agents affecting signaling activities relevant to GBM, for example, the testing of numerous molecules targeting receptor tyrosine kinase (RTK) kinase domains [9, 10].

The PI3K/Akt/mTOR signaling pathway, activated by growth factor RTKs, is known to be important in GBM progression and may be a promising pathway for targeted therapy [11]. The phosphatidylinositol-3-kinase (PI3K) pathway is commonly deregulated in human cancers including GBM [12]. PI3K translocates to the plasma membrane and catalyzes phosphatidylinositol 3, 4, 5-triphosphate (PIP3) production from phosphatidylinositol bisphosphate (PIP2) [13]. PIP3 activates serine/threonine kinase phosphoinositide-dependent kinase 1 (PDK1) and AKT, which leads to the suppression of apoptosis [14]. The high frequency of PI3K pathway alterations observed in GBM has spurred interest in the identification of novel modulators of this pathway.

Mammalian target of rapamycin (mTOR) is an important mediator of cellular responses to PI3K/Akt/mTOR signaling and has recently emerged as a compelling candidate target for GBM treatment. mTOR is a member of the serine/threonine protein kinase family and is a downstream target of PI3K. mTOR activation leads to cell growth, cell proliferation, and angiogenesis. mTOR signaling activity is dysregulated in certain solid tumors including GBM [15, 16]. mTOR is found as a component of two distinct complexes. The mTORC1 complex comprises mTOR, Raptor, mLST8, and PRAS40; this complex activates S6K1 and subsequently S6, leading to increased cell proliferation and growth [17]. mTORC2 comprises mTOR, Rictor, Sin1, and mLST8; its role is not well understood [18]. mTOR has been the target of several preclinical studies, including experiments using subcutaneous glioma models in which dual inhibition of both PI3K and mTOR was tested [19].

Testing of both PI3K and mTOR reflects a growing consensus that inhibition of individual molecular targets is unlikely to succeed as a therapeutic strategy for solid tumors. This thinking is based on greater understanding of the complexity of signaling activities underlying malignant transformation, as well as the ability of tumor cells to dynamically adapt to physiological stressors. It is increasingly clear that many of the most effective targeted cancer therapies owe their efficacy to unanticipated synergistic inhibition affecting multiple targets.

Autophagy is involved in numerous cellular stress responses including cell adaptation under nutrient deprivation and cell death. Autophagy involves the formation of autophagosomes containing damaged proteins and organelles, which are degraded by proteases following fusion with lysosomes. Autophagy plays dual regulatory roles influencing cell survival that affect the genesis and development of tumors [20]. In the early stages of some types of cancer, autophagy reportedly leads to the death of tumor cells and thus functions to maintain normal organismal homeostasis [21].

Taxifolin, also known as dihydroquercetin, is a flavanone found in milk thistle [22]. Among other biological effects, taxifolin exhibits antioxidant and anti-inflammatory properties [23]. Studies in colon cancer cells have identified taxifolin as a potential cancer chemopreventive agent that acts through an antioxidant response element- (ARE-) dependent mechanism [24]. Other studies reported that taxifolin directly inhibits kinase activities of EGFR and PI3K and exerts strong chemopreventive effects against UV-induced skin carcinogenesis [25].

We have investigated the dual inhibitory effects of taxifolin on mTOR and PI3K in GBM. We used *in silico*, *in vitro*, and *in vivo* approaches to demonstrate that taxifolin can act synergistically on mTOR and PI3K to promote autophagy and suppress lipid synthesis, thus inhibiting the growth of GBM. Our findings indicate that taxifolin has the potential to become a valuable drug for targeted molecular therapy.

## 2. Materials and Methods

### 2.1. Molecular Docking

Docking calculations were based on the previously determined structures of mTOR (PDB: 4jt5) [26] and PI3K (PDB: 2wxl) [27] using AutoDock 4.0 [28] with a Lamarckian genetic algorithm. To evaluate ligand-receptor binding energies, AutoGrid was used to generate a grid map of 80 × 80 × 80 points spaced at 0.375 Å. Docking parameters were set to 200 GA runs with an energy evaluation of 25,000,000. All remaining docking parameters were set to default values. Docked conformations were clustered using a tolerance of 2 Å for root mean square deviations (RMSDs) and ranked based on docking energies.

### 2.2. Molecular Dynamics Simulation

The Amber 14.0 simulation suite [29] was used for molecular dynamics (MD) simulations and data analysis. All-atom models of mTOR and PI3K were generated using the tleap module based on initial models. To resolve conflicting contacts among residues, energy minimization was performed by the steepest descent method for 500 steps followed by the conjugate gradient method for 500 steps. Protein was then solvated with water in a truncated tetrahedral periodic box (76.096 × 76.096 × 76.096 nm). The TIP3P [30] water model was used and five Na^+^ counterions were added to neutralize the system. Prior to the production phase, an equilibration protocol was applied as follows: first, the solvent was relaxed by energy minimization while restraining protein atomic positions with a harmonic potential. The system was then energy-minimized without restraints for 2,500 steps using a combination of steepest descent and conjugate gradient methods. The system was gradually heated from 0 to 300°K over 20 ps using the NVT ensemble. Finally, 5000 ps MD simulation was conducted at 1 ATM, 300°K, using the NPT ensemble. The SHAKE algorithm [31] was applied during the simulation to constrain covalent bonds to hydrogen atoms. A 2 fs time step and a nonbond interaction cutoff radius of 12.0 Å were used. Coordinates were saved every 1 ps throughout the process. AMBER ff03 [32] was used for protein and AMBER GAFF [33] for ligand. To develop parameters for terpinen-4-ol, the electrostatic potential of taxifolin was obtained from GAUSSIAN 2003 [34] set at HF/6-31G after geometric optimization at the same level. The partial charges were derived by fitting the gas-phase electrostatic potential using the restrained electrostatic potential (RESP) method [35]. The ligand missing interaction parameters were generated using antechamber tools in Amber. The long-range electrostatic parameters were calculated using the particle-mesh Ewald (PME) method [35]. Molecular mechanics generalized born surface area (MM-GBSA) was used to estimate binding energies. An AMD Opteron (tm) 192 Processor CPUs 2.0 GHz was used in simulation studies.

### 2.3. PI3K Kinase Inhibition Assay

The *in vitro* PI3K kinase assay has been described previously [36]. Briefly, active PI3K (100 ng) was incubated with taxifolin (0, 20, 40, or 80 mmol/L) or positive control LY294002 (20 mmol/L) for 10 min at 37°C. Phosphatidylinositol (0.5 mg/mL, MP Biomedicals) was added and the mixture was incubated for 5 min at room temperature (RT). This was followed by the addition of reaction buffer (10 mmol/L Tris-HCl (pH7.6), 60 mmol/L MgCl2, and 0.25 mmol/L ATP, 10 mCi *γ*-^32^PATP) and incubation for 10 min at 37°C. Termination buffer (1 part 4 N HCl, 3 parts 1 : 1 chloroform: methanol) was added to stop the reaction. After mixing, the lower (chloroform) phase was spotted onto a silica gel plate (Merck KGaA) and ^32^P-labeled phosphatidylinositol-3-phosphate (PIP3) was resolved by thin-layer chromatography and visualized by autoradiography.

### 2.4. mTOR Kinase Inhibition Assay

The mTOR kinase inhibition assay has been described previously [37]. Briefly, the assay was performed in 30 *μ*L of buffer (1 *μ*g PHAS-I, 120 mM NaCl, 40 mM HEPES, pH 7, 0.3% CHAPS, 4 mm MnCl_2_, 10 mM DTT, protease inhibitor (Sigma), 2 *μ*g/mL heparin, 100 *μ*m ATP, 2 *μ*Ci *γ*-32P ATP). Taxifolin (0, 20, 40, or 80 mmol/L) or PI-103 (20 mmol/L, positive control) was added and triplicate measurements were made for each concentration. The kinase reaction was terminated by spotting onto nitrocellulose, which was then washed several times with PBS. The radioactivity remaining on the nitrocellulose sheet was quantified by phosphorimaging. IC50 values were determined by fitting the data to a sigmoidal dose-response curve.

### 2.5. Cell Culture

Five human glioma cell lines (U87, LN229, SF188, A1207, and SF767) were obtained from the Chinese Academy of Sciences. The lines vary in mutational status at the PTEN or p53 loci, which are frequently inactivated in gliomas. The cell lines were maintained in Dulbecco's Modified Eagle Medium (DMEM) with 10% FBS at 37°C, 5% CO_2_ in a humidified incubator. Taxifolin (80 *μ*m) or PI-103(20 *μ*m) (in dimethyl sulfoxide, Sigma-Aldrich) was added to cells 24 hr prior to harvesting.

### 2.6. Western Blotting

Whole-cell lysates were prepared from cells collected in lysis buffer (50 mM Tris pH7.5, 150 mM NaCl, 1 mM EDTA, 1% NP40, 0.5% sodium deoxycholate, 1.0% SDS, 2 mM NaF, 2 mM Na_3_VO_4_, and protease inhibitors (Roche) followed by sonication and centrifugation at 13,000*g* for 10 minutes. Tumor lysates were prepared by homogenizing tumor tissue in lysis buffer and centrifuging at 13,000*g* for 10 minutes at 4°C. Proteins were quantified using a BCA assay kit, separated by electrophoresis on SDS PAGE gels, and transferred to PVDF membranes. After one hour in blocking buffer (SuperBlock), membranes were incubated overnight with primary antibodies. The following antibodies were used: anti-p-Akt (Cell Signaling), anti-Akt (Cell Signaling), anti-rpS6 (Abcam), anti-p-rpS6 (Abcam), anti-mTOR (Sigma-Aldrich), anti-p-mTOR (Sigma-Aldrich), anti-ERK (Abcam), anti-p-ERK (Abcam), anti-LC3B-II (Abcam), anti-Atg7 (Santa Cruz Biotechnology), anti-Atg12 (Santa Cruz Biotechnology), anti-Beclin-1 (Abcam), anti-C/EBP*α* (Abcam), anti-PPAR*γ* (Abcam), anti-FABP4 (Abcam), anti-FAS (Abcam), anti-Tubulin (Abcam), and anti-*β*-Actin (Santa Cruz Biotechnology). The anti-Tubulin and anti-*β*-Actin were used as controls. Specific protein bands were imaged using secondary antibody conjugated with horseradish peroxidase and chemiluminescent ECL reagents. Image J was used for quantification.

### 2.7. Cell Viability

Cell viability was determined using Cell Counting Kit-8 (Dojindo, Japan), which employs a redox assay similar to 3-(4, 5-dimethylthiazol-2-yl)-2, 5-diphenyltetrazolium bromide (MTT).

### 2.8. Animal Experiments

*In vivo* studies to evaluate drug effects on inhibition of tumor growth were performed using nude mice. 2 × 10^6^ U87 cells were subcutaneously transplanted into right and left flanks and tumor growth was monitored daily. Drug administration was initiated when the tumors reached a size of 100–120 mm^3^. Mice exhibiting no significant differences in tumor volume before drug treatment were sorted into 3 groups of 12 mice per group. Mice received once daily intraperitoneal (I.P.) administration of either 10% DMSO (vehicle control), taxifolin (100 mg/kg in 10% DMSO) or PI-103 (100 mg/kg in 10% DMSO). All mice were sacrificed under deep anesthesia (pentobarbital sodium, 40 mg/kg) after tumor size reached over 1 cm in diameter in the vehicle control group. Tumor volumes were calculated as (length ∗ width ∗ width/2). All tumors were dissected and weighted. The animal protocol was approved by Institutional Animal Care and Use Committee (IACUC).

### 2.9. Quantitative PCR

RNA was isolated using Total RNA Isolation Kit, and 1 *µ*g was reverse transcribed using Applied Biosystems reagents according to the manufacturer's instructions. cDNA was diluted 10-fold and quantified. Duplicate or triplicate RT-PCR reactions were performed for each sample using SYBR green and a QuantStudio 6 Flex real-time PCR system. Expression was normalized to the glyceraldehyde-3-phosphate dehydrogenase (GAPDH) gene. Primer sequences used for real-time RT-PCR are as follows (Table 1).

### 2.10. Statistical Analysis

Data are presented as mean ± SD. Student's *t*-test or one-way ANOVA was used for statistical analysis. *p* < 0.05 was considered significant.

## 3. Results

### 3.1. Taxifolin Binds to the Rapamycin Binding Site of mTOR

**Figure 1:**
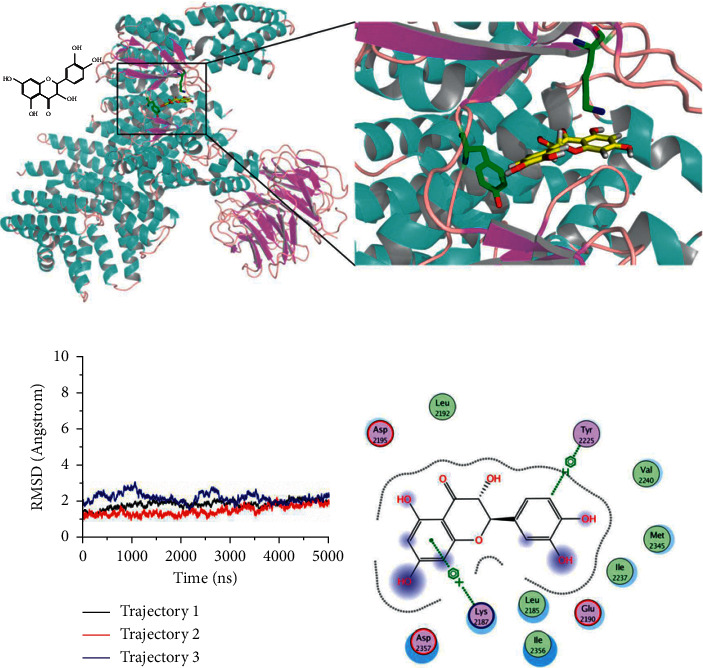
*In silico* analysis of taxifolin-mTOR interactions. (a) Conformation of taxifolin and mTOR during molecular dynamics simulation. (b) Root mean square deviations (RMSD) of the taxifolin-mTOR complex. (c) Interactions between taxifolin and mTOR.

Molecular dynamics simulations showed that taxifolin can bind to the rapamycin binding site of mTOR (Figure 1(a)). Taxifolin can have an arene-cation interaction with Lys^2187^ and an arene-H interaction with Tyr^2225^ (Figure 1(c)). The taxifolin-mTOR complex was stable. RMSDs of the complex remained between 1.5 and 3 Å in three replicate molecular dynamics simulations of 5000 ps (Figure 1(b)) and were invariant in the final 1000 ps (Figure 1(b)). These findings indicate that taxifolin can bind firmly to the key rapamycin binding site of mTOR and inhibit activity. In the 3-dimensional structure (Figure 1(a)), taxifolin is surrounded by amino acids around the binding pocket, which is also reflected in Figure 1(c).

### 3.2. Taxifolin Binds to the Active Site of PI3K

**Figure 2:**
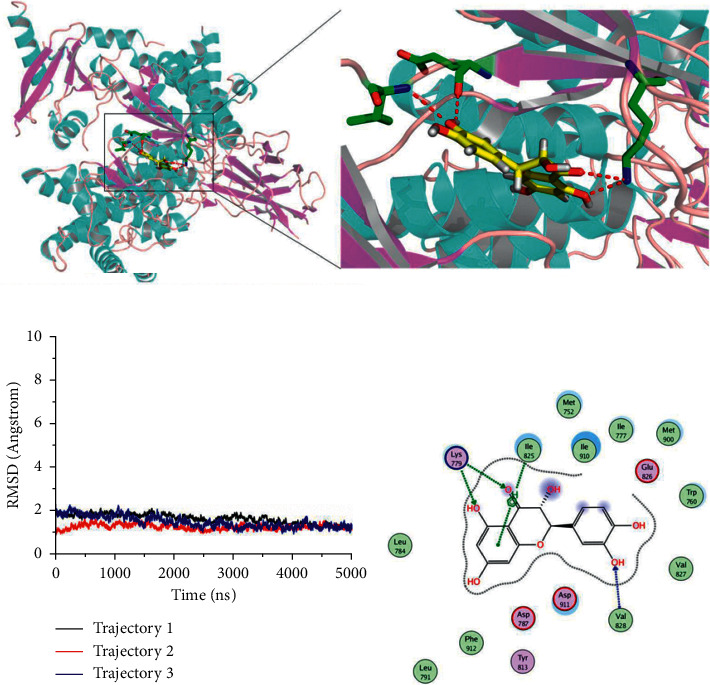
*In silico* analysis of taxifolin-PI3K interactions. (a) Conformation of taxifolin and PI3K during molecular dynamics simulation. Red line denotes hydrogen bond. (b) RMSD of the taxifolin-PI3K complex. (c) Interactions between taxifolin and PI3K.

We also used molecular dynamics simulations to analyze taxifolin and PI3K. As shown in Figure 2, taxifolin can bind to the active site of PI3K and form hydrogen bonds with Lys^779^, Ile^825^, and Val^828^ (Figure 2(a)). Taxifolin is surrounded by amino acids around the binding pocket (Figure 2(c)). The structure of the complex is stable. Throughout the entire 5000 ps simulation (Figure 2(b)), RMSDs remained between 1.5 and 2 Å, and from 3,000 ps, the RMSD values tended to remain stable, suggesting that taxifolin can firmly integrate into the pocket of PI3K.

The MM-GBSA method was used to analyze binding free energy during molecular dynamics simulations. The binding free energy of taxifolin and mTOR was −46.2, with van der Waals forces playing an important role. The binding free energy of taxifolin and PI3K was −38.9, with electrostatic interactions playing a major role. Altogether, these results indicate that taxifolin can stably bind both mTOR and PI3K.

### 3.3. Taxifolin Inhibits mTOR and PI3K Activities

**Figure 3:**
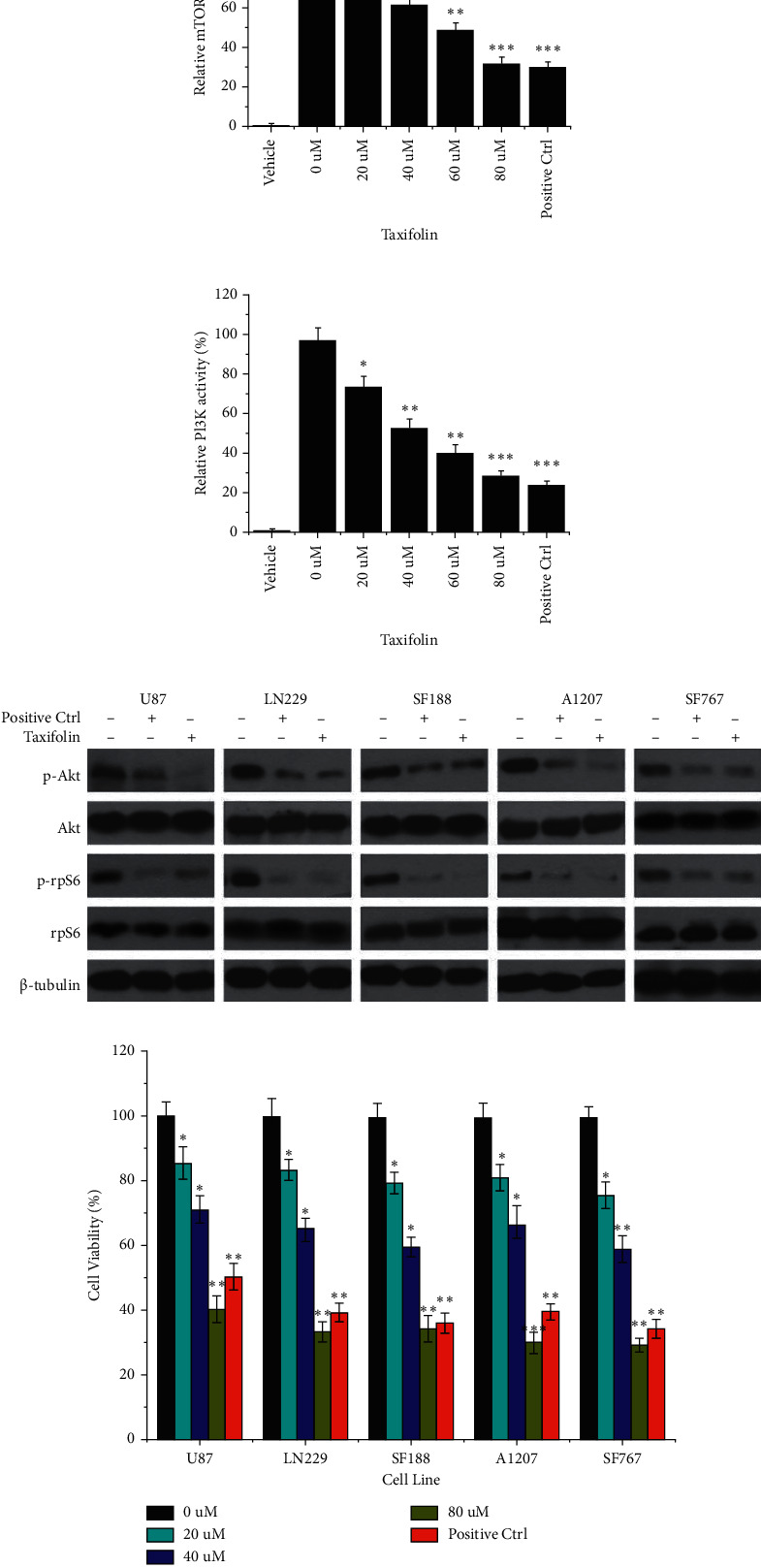
Taxifolin inhibition of mTOR and PI3K activity *in vitro*. (a) Relative activity of mTOR treated with taxifolin at various concentrations (0, 20 *μ*m, 40 *μ*m, 60 *μ*m, and 80 *μ*m). (b) Relative activity of PI3K treated with taxifolin at various concentrations (0, 20 *μ*m, 40 *μ*m, 60 *μ*m, and 80 *μ*m). (c) Western blot analysis of total protein from five glioma cell lines (U87, LN229, SF188, A1207, and SF767) treated with taxifolin. (d) Cell viability analysis of five glioma cell lines (U87, LN229, SF188, A1207, and SF767) treated with 0, 20 *μ*m, 40 *μ*m, and 80 *μ*m taxifolin. ^*∗*^*p* < 0.05, ^*∗∗*^*p* < 0.01, and ^*∗∗∗*^*p* < 0.001.

Based on our *in silico* findings, we proceeded to biochemical tests of whether taxifolin could act as a dual inhibitor of mTOR and PI3K. An mTOR kinase inhibition assay showed that mTOR activity decreased with increasing taxifolin concentration (Figure 3(a)). At 80 *µ*m taxifolin, mTOR activity was inhibited 68% compared to control. A PI3K kinase inhibition assay showed similar effects (Figure 3(b)). At 80 *µ*m taxifolin, the activity of PI3K was only 28.25% of control. Thus, biochemical studies confirm the results of *in silico* analysis.

We tested the effects of taxifolin on five different glioma cell lines: U87, LN229, SF188, A1207, and SF767. Phosphorylation of AKT and rpS6 is key events in the PI3K (Villanueva et al.) and mTOR [38] signaling pathways, respectively. Western blot analysis (Figure 3(c)) showed that treatment with taxifolin led to decreased levels of p-AKT with total AKT unchanged and decreased p-rpS6 without effect on total rpS6. The downregulation of p-AKT and p-rpS6 levels suggested that taxifolin effectively inhibited the activity of mTOR and PI3K *in vitro*. We furthermore observed that cell viability decreased with increasing concentrations of taxifolin (Figure 3(d)).

### 3.4. Taxifolin Could Inhibit Tumors In Vivo

**Figure 4:**
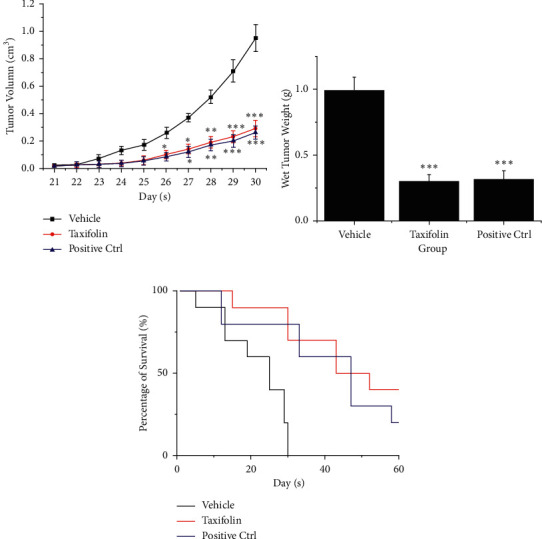
Taxifolin inhibition of GBM *in vivo*. (a) Tumor volume after treatment with vehicle (DMSO), taxifolin, or PI-103 (positive control) 21 to 30 days after inoculation of nude mice with U87MG glioma cells. (b) Tumor wet weight after treatment with vehicle, taxifolin, or PI-103. (c) Survival rate of mice treated with vehicle, taxifolin, or PI-103. ^*∗*^*p* < 0.05, ^*∗∗*^*p* < 0.01, and ^*∗∗∗*^*p* < 0.001.

We then investigated whether taxifolin could inhibit tumors *in vivo*. Mice inoculated in the armpits with the U87MG glioma cell line were administrated I.P. taxifolin or vehicle control. Tumor volumes were assessed from day 21 following tumor cell inoculation. As shown in Figure 4(a), the tumor volumes were significantly lower in the taxifolin group than in the vehicle control group. By day 30, the tumor volume was 0.29 cm^3^ in the taxifolin group but 0.95 cm^3^ in the vehicle control group. The tumor wet weight was also significantly lower in the taxifolin treatment group (Figure 4(b)). These findings showed that taxifolin effectively inhibited tumor growth *in vivo*. We also observed that the survival rate of mice was significantly increased as a result of taxifolin treatment (Figure 4(c)).

### 3.5. Taxifolin Inhibits mTOR and PI3K Pathway Activities in Tumor Cells

**Figure 5:**
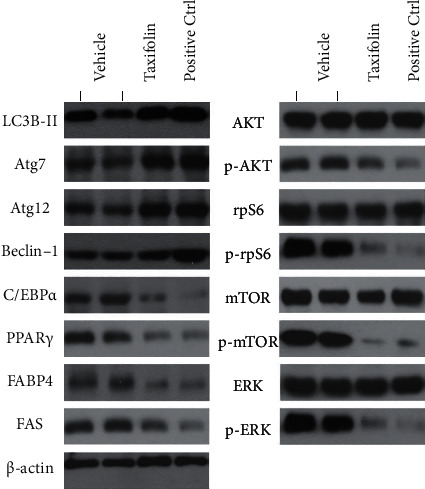
Taxifolin-induced *in vivo* inhibition of PI3K and mTOR signaling pathway activities, increased autophagy, and reduced lipid synthesis. Taxifolin treatment reduced levels of signaling pathway markers p-AKT, p-rpS6, p-mTOR, and p-ERK. Taxifolin treatment increased levels of autophagy markers LC3B-II, Atg7, atg12, and Beclin-1. Taxifolin treatment decreased levels of lipid synthesis markers C/EBP*α*, PPAR*γ*, FABP4, and FAS.

**Figure 6:**
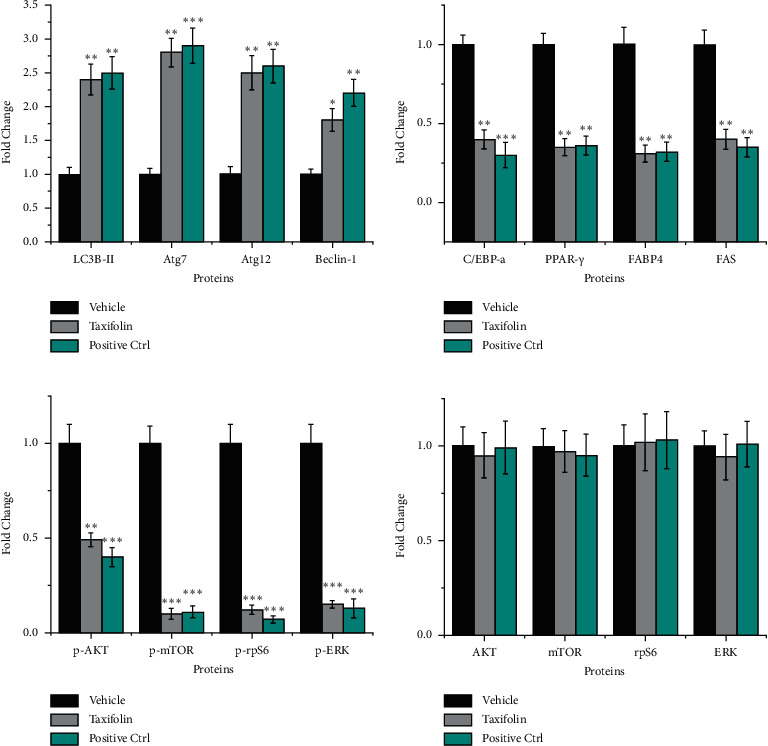
Quantification of western blotting results. (a) LC3B-II, Atg7, Atg12, and Beclin-1. (b) C/EBP*α*, PPAR*γ*, FABP4, and FAS. (c) p-AKT, p-mTOR, p-rpS6, and p-ERK. (d) AKT, mTOR, rpS6, and ERK. ^*∗*^*p* < 0.05, ^*∗∗*^*p* < 0.01, and ^*∗∗∗*^*p* < 0.001.

Western blot analysis was used to investigate the effects of taxifolin on mTOR and PI3K pathway activities in tumor cells. As shown in Figures 5 and 6, taxifolin treatment led to decreased levels of p-AKT, p-rpS6, p-ERK, and p-mTOR while total AKT, rpS6, ERK, and mTOR levels were unchanged. Thus, taxifolin associated inhibition of tumor growth was correlated with inhibition of mTOR and PI3K pathway activity.

### 3.6. Taxifolin Promoted the Expression of Autophagy-Related Genes

**Figure 7:**
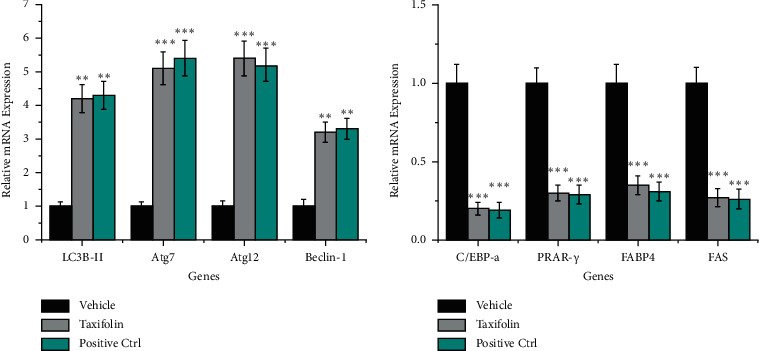
Taxifolin treatment led to increased levels of autophagy-related gene expression and decreased levels of lipid synthesis-related gene expression as determined by qPCR. ^*∗*^*p* < 0.05, ^*∗∗*^*p* < 0.01, and ^*∗∗∗*^*p* < 0.001.

Because it is known that activation of mTOR can inhibit autophagy [39], we also examined the expression of key autophagy pathway related proteins LC3B-II, Atg7, atg12, and Beclin-1 (Glick et al. [40]) in tumors treated with taxifolin. We found that levels of LC3B-II, Atg7, atg12, and Beclin-1 proteins were increased in tumors of mice treated with taxifolin compared to vehicle control. qPCR analysis confirmed that LC3B-II, Atg7, atg12, and Beclin-1 mRNA levels were increased after taxifolin treatment (Figure 7(a)). Our results indicate that autophagy was activated in tumor cells and suggest that taxifolin-induced inhibition of mTOR increased autophagy activity *in vivo*.

### 3.7. Taxifolin Inhibited the Expression of Lipid Synthesis-Related Genes

In GBM, lipid metabolism is abnormal and lipid synthesis is enhanced. mTOR is known to play an important role in cellular lipid metabolism [41]. mTOR activation leads to increased lipid metabolism. We, therefore, investigated whether taxifolin treatment inhibited lipid synthesis in tumors. We found that expression of C/EBP*α*, PPAR*γ*, FABP4, and FAS, four key proteins involved in lipid synthesis [42], was significantly downregulated in tumors from mice treated with taxifolin. qPCR analysis confirmed that C/EBP*α*, PPAR*γ*, FABP4, and FAS mRNA levels were decreased as a result of taxifolin treatment (Figure 7(b)). Thus, taxifolin-induced inhibition of tumor growth was associated with lower expression of genes important for lipid synthesis.

## 4. Discussion

We took *in silico*, biochemical, *in vitro* cell culture, and *in vivo* tumor model approaches to demonstrate that the flavonoid molecule taxifolin can inhibit mTOR and PI3K signaling, promote autophagy, and inhibit lipid synthesis in GBM tumor cells. Taxifolin inhibited the growth of human glioma cell lines in culture and GBM *in vivo*. These observations point to taxifolin as a potential drug for use in treating GBM.

Single target therapies are limited in that they address only one aspect of a disease process. Multitargeting drugs have the potential advantage of eliciting synergistic effects, especially for cancer [43]. As targeted therapies have progressed, multiple PI3K/mTOR dual inhibitors have entered clinical trials and have shown good therapeutic effects [44–47]. We present evidence that taxifolin is a promising dual-targeting molecule that can inhibit both mTOR and PI3K. Taxifolin is a naturally occurring flavonoid that can be extracted from milk thistle. Moreover, its toxicity is very low [48, 49]; studies have shown no obvious damage to the liver and kidneys in mice.

Computer-aided drug design (CADD) has increasingly been used for targeted drug screening and design [50]. CADD offers advantages of savings in terms of both costs and time. It can be used to rapidly predict the binding sites of candidate molecules on target proteins [51, 52]. More importantly, it can be used to investigate specific binding mechanisms [53]. Here, we showed using molecular dynamics simulation that taxifolin can bind to the rapamycin site of mTOR involving arene-cation interaction with Lys^2187^ and arene-H interaction with Tyr^2225^. Taxifolin can also bind to the catalytic core site of PI3K (p110*α*) and form hydrogen bonds with Lys^779^, Ile^825^, and Val^828^. The binding is predicted to be stable in both cases and these data predict potential inhibition of mTOR and PI3K activity by taxifolin.

PI3K plays an important role in GBM tumorigenesis and growth [54]. Key factors in the PI3K-Akt signaling pathway such as p110*α* are related to cellular transformation [55]. Taxifolin has been shown to effectively inhibit the activity of p110*α*. As an important downstream molecule of the PI3K pathway, mTOR is also important for GBM [37]. mTOR is a key regulator of cell growth and proliferation, modulating effects of diverse stimuli including growth factors and nutrients [56]. This pathway is often found to be overactivated in GBM, promoting cell transformation and tumor progression [57]. Here, we describe experiments demonstrating that taxifolin can bind to the key site of mTOR, thereby inhibiting mTOR activity and eliciting antitumor effects.

mTOR is a Ser/Thr kinase involved in multiple processes including cell differentiation, ribosome production, and metabolic regulation. Of particular interest here, mTOR plays a key role in regulating autophagy [58]. Autophagy is inhibited as a result of mTOR pathway activation. mTOR is found in rapamycin-sensitive mTORC1 and rapamycin-insensitive mTORC2 complexes. The ULK1-ATG13-RB1CC1-C12orf44/Atg101 complex phosphorylates mTORC1 and negatively regulates the formation of autophagosomes, reflecting the level of autophagy [59]. Autophagy can lead to type II programmed cell death and evidence suggests that autophagy may be important in the treatment of GBM [60, 61]. Autophagy promoting drugs, such as temozolomide, can selectively kill apoptosis-resistant glioblastoma cells [62, 63]. In this study, we found that treatment with taxifolin increased the expression of autophagy-related proteins including LC3B-II, Atg7, atg12, and Beclin-1 suggesting that the autophagic pathway was activated in our tumor model.

mTOR is also closely associated with lipid metabolism [41]. mTORC1 is activated when there is sufficient energy to promote glycolysis, nucleic acid synthesis, and glutamine metabolism and the mTORC1 pathway promoting anabolism in tumors is often found overactivated in GBM. By contrast, the AMPK pathway promoting catabolism is often found inhibited [64]. This configuration promotes rapid cell proliferation and transforms cellular metabolism to a tumor profile. Altered lipid synthesis is another important metabolic change characteristic of GBM [65]. Fatty acid synthesis is overactive in tumors compared to precancerous lesions. Fatty acids can be used to synthesize tumor-promoting lipid signaling molecules. Effectively suppressing lipid synthesis in tumor cells can therefore inhibit tumor growth. In our tumor model, we found that taxifolin treatment led to reduced expression of genes encoding fatty acid synthesis-related proteins C/EBPa, PPAR*γ*, FABP4, and FAS, suggesting that tumor cell lipid synthesis was reduced and that this may have contributed to the inhibition of tumor growth.

## 5. Conclusions

In summary, we show that taxifolin is a dual-target inhibitor of mTOR and PI3K and represents a potential drug for treating GBM. Based on these results, it may be fruitful to conduct screens for additional dual-target inhibitors. It is not yet known whether taxifolin-induced inhibition of GBM may be mediated through effects on additional pathways. Nevertheless, our observations indicate that further investigations are warranted to confirm taxifolin's potential as a treatment for GBM.

## Figures and Tables

**Table 1:** RT-PCR primers.

Gene	Forward	Reverse
LC3B-II	GAGAAGCAGCTTCCTGTTCTGG	GTGTCCGTTCACCAACAGGAAG
ATG7	CGTTGCCCACAGCATCATCTTC	CACTGAGGTTCACCATCCTTGG
ATG12	GGGAAGGACTTACGGATGTCTC	AGGAGTGTCTCCCACAGCCTTT
C/EBP-*α*	AGGAGGATGAAGCCAAGCAGCT	AGTGCGCGATCTGGAACTGCAG
PPAR-*γ*	TCGGCGAGGATAGTTCTGGAAG	GACCACAGGATAAGTCACCGAG
FABP4	ACGAGAGGATGATAAACTGGTGG	GCGAACTTCAGTCCAGGTCAAC
FAS	GGACCCAGAATACCAAGTGCAG	GTTGCTGGTGAGTGTGCATTCC
Beclin-1	CTGGACACTCAGCTCAACGTCA	CTCTAGTGCCAGCTCCTTTAGC
GAPDH	GTCTCCTCTGACTTCAACAGCG	ACCACCCTGTTGCTGTAGCCAA

## Data Availability

The data that support the findings of this study are available from the corresponding author upon reasonable request.

## References

[1] Le Rhun E., Preusser M., Roth P. (2019). Molecular targeted therapy of glioblastoma. *Cancer Treatment Reviews*.

[2] Guan X., Hasan M. N., Maniar S., Jia W., Sun D. (2018). Reactive astrocytes in glioblastoma multiforme. *Molecular Neurobiology*.

[3] Zottel A., Šamec N., Videtič Paska A., Jovčevska I. (2020). Coding of glioblastoma progression and therapy resistance through long noncoding RNAs. *Cancers*.

[4] Iser I. C., Pereira M. B., Lenz G., Wink M. R. (2017). The epithelial-to-mesenchymal transition-like process in glioblastoma: an updated systematic review and in silico investigation. *Medicinal Research Reviews*.

[5] Alifieris C., Trafalis D. T. (2015). Glioblastoma multiforme: pathogenesis and treatment. *Pharmacology & Therapeutics*.

[6] Ali J. S., Ashford J. M., Swain M. A. (2021). Predictors of cognitive performance among infants treated for brain tumors: findings from a multisite, prospective, longitudinal trial. *Journal of Clinical Oncology*.

[7] Quail D. F., Bowman R. L., Akkari L. (2016). The tumor microenvironment underlies acquired resistance to CSF-1R inhibition in gliomas. *Science*.

[8] Dunn-Pirio A. M., Vlahovic G. (2017). Immunotherapy approaches in the treatment of malignant brain tumors. *Cancer*.

[9] Li Chew C., Lunardi A., Gulluni F. (2015). In vivo role of INPP4B in tumor and metastasis suppression through regulation of PI3K-AKT signaling at endosomes. *Cancer Discovery*.

[10] Bastien J. I. L., McNeill K. A., Fine H. A. (2015). Molecular characterizations of glioblastoma, targeted therapy, and clinical results to date. *Cancer*.

[11] Alzahrani A. S. (2019). PI3K/Akt/mTOR inhibitors in cancer: at the bench and bedside. *Seminars in Cancer Biology*.

[12] Akinleye A., Avvaru P., Furqan M., Song Y., Liu D. (2013). Phosphatidylinositol 3-kinase (PI3K) inhibitors as cancer therapeutics. *Journal of Hematology & Oncology*.

[13] Zhao W., Qiu Y., Kong D. (2017). Class I phosphatidylinositol 3-kinase inhibitors for cancer therapy. *Acta Pharmaceutica Sinica B*.

[14] Carnero A. (2010). The PKB/AKT pathway in cancer. *Current Pharmaceutical Design*.

[15] Janku F., Yap T. A., Meric-Bernstam F. (2018). Targeting the PI3K pathway in cancer: are we making headway?. *Nature Reviews Clinical Oncology*.

[16] Ortega M. A., Fraile-Martínez O., Asúnsolo Á., Buján J., García-Honduvilla N., Coca S. (2020). Signal transduction pathways in breast cancer: the important role of PI3K/Akt/mTOR. *Journal of oncology*.

[17] Saxton R. A., Sabatini D. M. (2017). mTOR signaling in growth, metabolism, and disease. *Cell*.

[18] Oh W. J., Jacinto E. (2011). mTOR complex 2 signaling and functions. *Cell Cycle*.

[19] Bagci-Onder T., Wakimoto H., Anderegg M., Cameron C., Shah K. (2011). A dual PI3K/mTOR inhibitor, PI-103, cooperates with stem cell-delivered TRAIL in experimental glioma models. *Cancer Research*.

[20] Liu C., Xu P., Chen D. (2013). Roles of autophagy-related genes beclin-1 and LC3 in the development and progression of prostate cancer and benign prostatic hyperplasia. *Biomedical Reports*.

[21] Mizushima N., Komatsu M. (2011). Autophagy: renovation of cells and tissues. *Cell*.

[22] Zu Y., Wu W., Zhao X. (2014). Enhancement of solubility, antioxidant ability and bioavailability of taxifolin nanoparticles by liquid antisolvent precipitation technique. *International Journal of Pharmaceutics*.

[23] Zhang H., Tsao R. (2016). Dietary polyphenols, oxidative stress and antioxidant and anti-inflammatory effects. *Current Opinion in Food Science*.

[24] Weidmann A. E. (2012). Dihydroquercetin: more than just an impurity?. *European Journal of Pharmacology*.

[25] Sunil C., Xu B. (2019). An insight into the health-promoting effects of taxifolin (dihydroquercetin). *Phytochemistry*.

[26] Yang H., Rudge D. G., Koos J. D., Vaidialingam B., Yang H. J., Pavletich N. P. (2013). mTOR kinase structure, mechanism and regulation. *Nature*.

[27] Berndt A., Miller S., Williams O. (2010). The p110*δ* structure: mechanisms for selectivity and potency of new PI(3)K inhibitors. *Nature Chemical Biology*.

[28] Morris G. M., Goodsell D. S., Halliday R. S. (1998). Automated docking using a lamarckian genetic algorithm and an empirical binding free energy function. *Journal of Computational Chemistry*.

[29] Babin V., Berryman J. T. (2014). AMBER 14; university of California: San Francisco.

[30] Jorgensen W. L., Chandrasekhar J., Madura J. D., Impey R. W., Klein M. L. (1983). Comparison of simple potential functions for simulating liquid water. *The Journal of Chemical Physics*.

[31] Ryckaert J.-P., Ciccotti G., Berendsen H. J. C. (1977). Numerical integration of the cartesian equations of motion of a system with constraints: molecular dynamics of n-alkanes. *Journal of Computational Physics*.

[32] Duan Y., Wu C., Chowdhury S. (2003). A point-charge force field for molecular mechanics simulations of proteins based on condensed-phase quantum mechanical calculations. *Journal of Computational Chemistry*.

[33] Wang J., Wolf R. M., Caldwell J. W., Kollman P. A., Case D. A. (2004). Development and testing of a general amber force field. *Journal of Computational Chemistry*.

[34] Frisch M. J., Trucks G. W., Schlegel H. B. (2004). *Gaussian 03, revision C.02*.

[35] Bayly C. I., Cieplak P., Cornell W., Kollman P. A. (1993). A well-behaved electrostatic potential based method using charge restraints for deriving atomic charges: the RESP model. *The Journal of Physical Chemistry*.

[36] Kwon J. Y., Lee K. W., Kim J.-E. (2009). Delphinidin suppresses ultraviolet B-induced cyclooxygenases-2 expression through inhibition of MAPKK4 and PI-3 kinase. *Carcinogenesis*.

[37] Fan Q.-W., Knight Z. A., Goldenberg D. D. (2006). A dual PI3 kinase/mTOR inhibitor reveals emergent efficacy in glioma. *Cancer Cell*.

[38] Villanueva A., Chiang D. Y., Newell P. (2008). Pivotal role of mTOR signaling in hepatocellular carcinoma. *Gastroenterology*.

[39] Pattingre S., Espert L., Biard-Piechaczyk M., Codogno P. (2008). Regulation of macroautophagy by mTOR and beclin 1 complexes. *Biochimie*.

[40] Glick D., Barth S., Macleod K. F. (2010). Autophagy: cellular and molecular mechanisms. *The Journal of Pathology*.

[41] Caron A., Richard D., Laplante M. (2015). The roles of mTOR complexes in lipid metabolism. *Annual Review of Nutrition*.

[42] Agnihotri S., Zadeh G. (2015). Metabolic reprogramming in glioblastoma: the influence of cancer metabolism on epigenetics and unanswered questions. *Neuro-Oncology*.

[43] Raghavendra N. M., Pingili D., Kadasi S., Mettu A., Prasad S. V. U. M. (2018). Dual or multi-targeting inhibitors: the next generation anticancer agents. *European Journal of Medicinal Chemistry*.

[44] Wright S. C. E., Vasilevski N., Serra V., Rodon J., Eichhorn P. J. A. (2021). Mechanisms of resistance to PI3K inhibitors in cancer: adaptive responses, drug tolerance and cellular plasticity. *Cancers*.

[45] Rodon J., Dienstmann R., Serra V., Tabernero J. (2013). Development of PI3K inhibitors: lessons learned from early clinical trials. *Nature Reviews Clinical Oncology*.

[46] Wander S. A., Hennessy B. T., Slingerland J. M. (2011). Next-generation mTOR inhibitors in clinical oncology: how pathway complexity informs therapeutic strategy. *Journal of Clinical Investigation*.

[47] Kołodziej P., Nicoś M., Krawczyk P. A. (2021). The correlation of mutations and expressions of genes within the PI3K/Akt/mTOR pathway in breast cancer-a preliminary study. *International Journal of Molecular Sciences*.

[48] Abenavoli L., Izzo A. A., Milić N., Cicala C., Santini A., Capasso R. (2018). Milk thistle (*Silybum marianum*): a concise overview on its chemistry, pharmacological, and nutraceutical uses in liver diseases. *Phytotherapy Research*.

[49] Hackett E. S., Twedt D. C., Gustafson D. L. (2013). Milk thistle and its derivative compounds: a review of opportunities for treatment of liver disease. *Journal of Veterinary Internal Medicine*.

[50] Vucicevic J., Nikolic K., Mitchell J. B. O. (2019). Rational drug design of antineoplastic agents using 3D-QSAR, cheminformatic, and virtual screening approaches. *Current Medicinal Chemistry*.

[51] Kapetanovic I. M. (2008). Computer-aided drug discovery and development (CADDD): in silico-chemico-biological approach. *Chemico-Biological Interactions*.

[52] Ban F., Dalal K., Li H., LeBlanc E., Rennie P. S., Cherkasov A. (2017). Best practices of computer-aided drug discovery: lessons learned from the development of a preclinical candidate for prostate cancer with a new mechanism of action. *Journal of Chemical Information and Modeling*.

[53] Kitchen D. B., Decornez H., Furr J. R., Bajorath J. (2004). Docking and scoring in virtual screening for drug discovery: methods and applications. *Nature Reviews Drug Discovery*.

[54] Cloughesy T. F., Cavenee W. K., Mischel P. S. (2014). Glioblastoma: from molecular pathology to targeted treatment. *Annual Review of Pathology: Mechanisms of Disease*.

[55] Aoki M., Fujishita T. (2017). Oncogenic roles of the PI3K/AKT/mTOR axis. *Current Topics in Microbiology and Immunology*.

[56] Laplante M., Sabatini D. M. (2012). mTOR signaling in growth control and disease. *Cell*.

[57] Eckerdt F. D., Bell J. B., Gonzalez C. (2020). Combined PI3K*α*-mTOR targeting of glioma stem cells. *Scientific Reports*.

[58] Chiarini F., Evangelisti C., McCubrey J. A., Martelli A. M. (2015). Current treatment strategies for inhibiting mTOR in cancer. *Trends in Pharmacological Sciences*.

[59] Romero Y., Bueno M., Ramirez R. (2016). mTORC 1 activation decreases autophagy in aging and idiopathic pulmonary fibrosis and contributes to apoptosis resistance in IPF fibroblasts. *Aging Cell*.

[60] Lefranc F., Facchini V., Kiss R. (2007). Proautophagic drugs: a novel means to combat apoptosis‐resistant cancers, with a special emphasis on glioblastomas. *The Oncologist*.

[61] Pawlowska E., Szczepanska J., Szatkowska M., Blasiak J. (2018). An interplay between senescence, apoptosis and autophagy in glioblastoma multiforme-role in pathogenesis and therapeutic perspective. *International Journal of Molecular Sciences*.

[62] Villamañan L., Martínez-Escardó L., Arús C., Yuste V. J., Candiota A. P. (2021). Successful partnerships: exploring the potential of immunogenic signals triggered by TMZ, CX-4945, and combined treatment in GL261 glioblastoma cells. *International Journal of Molecular Sciences*.

[63] Oliveira K. A., Dal-Cim T. A., Lopes F. G., Nedel C. B., Tasca C. I. (2017). Guanosine promotes cytotoxicity via adenosine receptors and induces apoptosis in temozolomide-treated A172 glioma cells. *Purinergic Signalling*.

[64] Shackelford D. B., Shaw R. J. (2009). The LKB1-AMPK pathway: metabolism and growth control in tumour suppression. *Nature Reviews Cancer*.

[65] Guo D., Bell E. H., Chakravarti A. (2013). Lipid metabolism emerges as a promising target for malignant glioma therapy. *CNS Oncology*.

